# Pentatricopeptide repeat 153 (PPR153) restores maize C-type cytoplasmic male sterility in conjunction with RF4

**DOI:** 10.1371/journal.pone.0303436

**Published:** 2024-07-10

**Authors:** Jennifer S. Jaqueth, Bailin Li, Erik Vollbrecht

**Affiliations:** 1 Corteva Agriscience™, Johnston, IA, United States of America; 2 Department of Genetics, Development and Cell Biology, Iowa State University, Ames, IA, United States of America; Institute of Agricultural Resources and Regional Planning, Chinese Academy of Agricultural Sciences, CHINA

## Abstract

Maize (*Zea mays* L.) C-type cytoplasmic male sterility (CMS-C) is a highly used CMS system for maize commercial hybrid seed production. *Rf4* is the major dominant restorer gene for CMS-C. Inbreds were recently discovered which contain the restoring *Rf4* allele yet are unable to restore fertility due to the lack of an additional gene required for *Rf4*’s restoration. To find this additional gene, QTL mapping and positional cloning were performed using an inbred that contained *Rf4* but was incapable of restoring CMS-C. The QTL was mapped to a 738-kb interval on chromosome 2, which contains a Pentatricopeptide Repeat (PPR) gene cluster. Allele content comparisons of the inbreds identified three potential candidate genes responsible for fertility restoration in CMS-C. Complementation via transformation of these three candidate genes showed that *PPR153* (*Zm00001eb114660*) is required for *Rf4* to restore fertility to tassels. The *PPR153* sequence is present in B73 genome, but it is not capable of restoring CMS-C without *Rf4*. Analysis using NAM lines revealed that *Rf4* requires the presence of *PPR153* to restore CMS-C in diverse germplasms. This research uncovers a major CMS-C genetic restoration pathway and can be used for identifying inbreds suitable for maize hybrid production with CMS-C cytoplasm.

## Introduction

Cytoplasmic male sterility, CMS, is a maternally inherited trait found in at least 140 plant species caused by an incompatibility between the mitochondrial and nuclear genomes resulting in the failure to produce functional pollen [1]. CMS usually results from expression of a novel chimeric mitochondrial open reading frame (ORF) derived from combinations of mitochondrial gene-coding and noncoding sequences [2]. Tassel fertility can be restored by an array of nucleus encoded *Restorer of fertility* (*Rf*) genes, which function to suppress the effects of the sterility-causing CMS ORF. These CMS systems can be used in hybrid seed production once the germplasm has been characterized for the presence of *Rf* genes and their gene interactors.

Frequently, these *Rf* genes encode pentatricopeptide repeat (PPR) proteins, a class of sequence-specific RNA-binding proteins involved in post-transcriptional processing in the mitochondria and chloroplast [3,4]. PPRs are one of the largest protein families in land plants numbering 400 to over 1000 PPRs in each species [5]. The maize genome is predicted to contain 521 PPR genes [6].

Three major types of cytoplasm have been defined in maize: T (Texas), S (USDA) and C (Charrua) based on the DNA sequences of the mitochondria and can be distinguished by the nuclear restorer genes which function to counteract the sterility-inducting factors [7,8]. CMS-C is a widely used CMS system in maize seed production due to its stability across environments and its lack of disease susceptibility. CMS-C’s sterility-inducing factor is the mitochondrial chimeric *atp6c* gene, which contains a 481 bp leader sequence different than *atp6* [9,10]. The most commonly used CMS-C restorer gene, *Rf4* (*Zm00001eb332170*) on chromosome 8, encodes a bHLH transcription factor and is also annotated as *Male Sterile23*, which has an essential function in differentiation of the anther tapetal cells [11,12]. The restoring *Rf4* allele contains a Y187F substitution which controls restoration [12,13]. *Rf4* is nuclear-localized, and single cell RNA sequencing suggests *Rf4* may function in redox homeostasis in CMS-C pollen [14]. Previously it was thought that *Rf4* restored pollen function in all inbred backgrounds [15], although Liu et al. recently reported that *Rf4* failed to restore in a few Chinese inbreds [13]. Any inconsistency of the *Rf4* allele’s prediction of restoring ability can compromise the deployment of CMS-C in commercial hybrid seed production.

Because most previously identified nuclear restorer genes in plant CMS systems were found to encode proteins predicted to be targeted to the mitochondria, and most of them were also RNA-interacting PPR proteins, it was surprising when the CMS-C-restoring *Rf4* allele was identified as a variant of the MS23 locus, which encodes a nuclear-targeted transcription factor essential for anther development [11,12]. This finding suggested that the Rf4-ms23 allele is involved in regulating another gene whose product is mitochondrially targeted to modify the expression of the CMS-causative gene within the mitochondrion.

In this paper, we describe our process to identify a locus, *PPR153*, that is required for *Rf4* to restore CMS-C to fertility. We first examined two inbreds which contain the restoring *Rf4* allele yet were incapable of restoring CMS-C sterility. Using QTL mapping, we mapped the loci causing *Rf4*’s restoration failure to a single PPR cluster on chromosome 2. This cluster contains multiple restorers of fertility for other maize CMS systems, e.g., *Rf8* and Rf* for CMS-T, *Rf3* for CMS-S. Positional cloning and gene-content comparisons identified three possible candidate PPR genes involved in *Rf4* restoration, then transgenic complementation tests were used to identify the causal gene. The allele frequency of the validated gene was studied in the Nested Association Mapping (NAM) diverse founder lines, and the NAM inbreds were used to further explore the interaction between *Rf4* and this newly discovered PPR gene involved in CMS-C restoration.

## Results

### *Rf4* fails to restore CMS-C fertility in some inbreds but recovers functionality in F_1_ crosses

**Fig 1:**
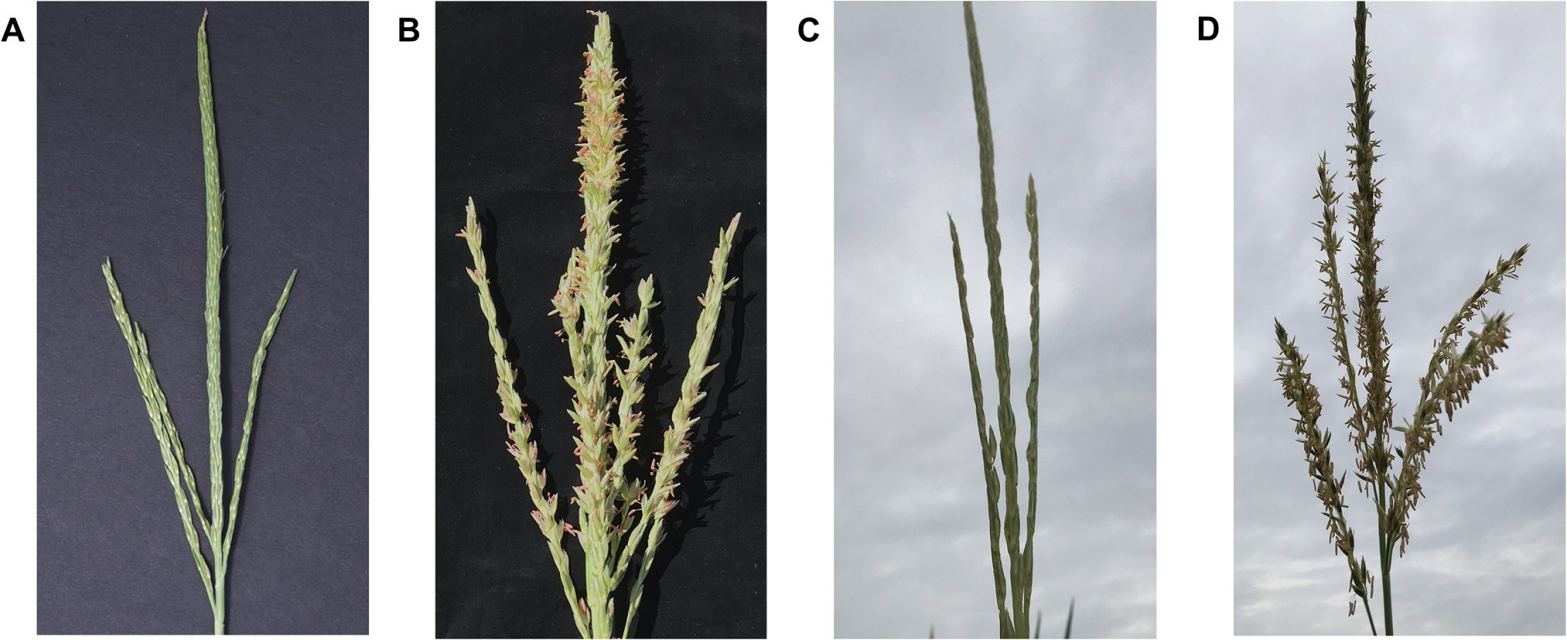
Tassel fertility of CMS-C inbreds and F_1_ crosses. (A) CMS-C line C-PH269A^*Rf4Rf4*^ has sterile tassels. (B) F_1_ cross C-PH269A ^*Rf4Rf4*^ x N-PH2FP0 ^*Rf4Rf4*^ has fertile tassels. (C) CMS-C line C-PH2F3V^*Rf4Rf4*^ has sterile tassels. (D) F_1_ cross C-PH2F3V^*Rf4Rf4*^ x N-PH480C ^*Rf4Rf4*^ has fertile tassels.

An inbred used in hybrid seed production, C-PH7HG ^*Rf4Rf4*^, was converted to CMS-C cytoplasm. Despite containing the restoring *Rf4* allele, C-PH7HG ^*Rf4Rf4*^ had sterile tassels when grown in multiple locations across North America, South America and Europe. Four inbreds derived from PH7HG also contained restoring *Rf4* and showed the unexpected sterile phenotype when converted to CMS-C cytoplasm. Two of these inbreds, C-PH269A and C-PH2F3V, were confirmed to contain the restoring *Rf4* F187 allele through resequencing and then were used for mapping studies. In 2018 in Johnston IA, C-PH269A^*Rf4Rf4*^ and C-PH2F3V^*Rf4Rf4*^ were grown in the field and had non-restored, sterile tassels despite containing restoring *Rf4* (Fig 1A and 1C). Two F_1_ crosses, C-PH269A^*Rf4Rf4*^ x N-PH2FP0 ^*Rf4Rf4*^ and C-PH2F3V^*Rf4Rf4*^ x N-PH480C ^*Rf4Rf4*^, were also phenotyped in the field for tassel fertility. Both F_1_’s had fully fertile tassels (Fig 1B and 1D).

### *Rf4*’s restoration failure mapped to a 783-kb interval on chromosome 2

**Fig 2:**
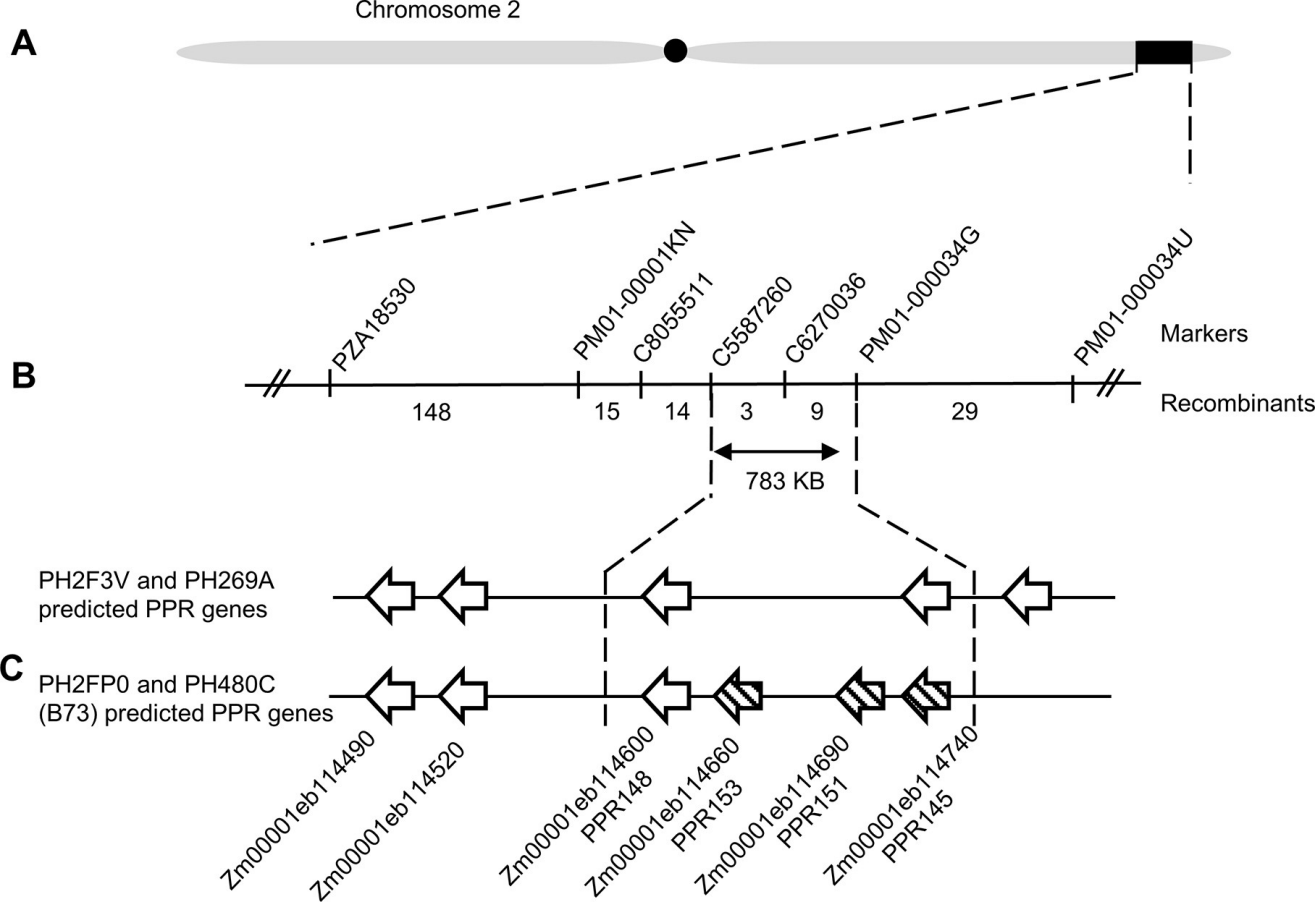
Positional cloning recombinants and gene content within fine mapping interval. (A) Region on chromosome 2 identified by whole genome QTL mapping which encompasses a PPR gene cluster containing known maize Rf genes. (B) The number of recombinants in each marker interval from the combined fine mapping populations. (C) PPRs located within the gene clusters. PPR names were collected from maizegdb.org as cataloged by Wei and Han 2016. The three candidate genes in the complementation test are indicated with hashed lines.

A C-PH269A^*Rf4Rf4*^ x (N-PH2FP0^*Rf4Rf4*^ x N-PH269A^*Rf4Rf4*^) BC_1_F_1_ mapping population of 152 individuals was created with PH269A^*Rf4Rf4*^ used as the recurrent parent. The tassels of the population were phenotyped on a 1 to 5 degree of fertility scale and did not significantly deviate from a ratio of 1:1 (χ2  = 0.105; p  = 0.746). This suggests that a single restoration gene is found within PH2FP0 (Table 1). QTL mapping identified one large effect QTL on chromosome 2 between left flanking marker PZA18530 and right flanking marker PM01-000034U with a LOD of 54 explaining 81% of phenotypic variation (Figs 2A and S1). The haplotype of PH269A at the QTL was associated with sterile tassels, and the haplotype of PH2FP0 at the QTL was associated with fertile tassels.

**Table 1:** Degree of tassel fertility in C-PH269A BC_1_F_1_ mapping population. χ^2^ test for Mendelian segregation of sterile to fertile (1:1). Scores of 1 and 2 are considered functionally sterile and 3 to 5 are considered functionally fertile.

Population	Total Plants	1	2	3	4	5	χ^2^	*p*-value
C-PH269A BC_1_F_1_	152	60	14	4	2	72	0.11	0.746

In 2019, 188 plants of the C-PH269A population and 291 plants of the C-PH2F3V population were phenotyped and genotyped with KASP markers designed within the mapping interval. Using a positional cloning approach, the mapping interval was determined to be between C8055511 and PM01-000034G, with an interval size of 1.39-Mb on the Zm-B73-REFERENCE-NAM-5.0 (S2 Fig). In 2020, 183 recombinant individuals were selected from a large C-PH2F3V BC1F2 population and were then phenotyped and genotyped. The mapping interval was reduced to 783-kb on the Zm-B73-REFERENCE-NAM-5.0, with a left flanking marker of C5587260 and right flanking marker of PM01-000034G. Marker C6270036 was co-segregating with the trait (Figs 2B and S2). PH2FP0 and PH480C contributed the restoring haplotypes within the chromosome 2 interval.

In 2021, F1 allelism crosses created with PH2F3V were used to evaluate restoring ability in multiple genetic backgrounds (Table 2). Four CMS-C sterile inbreds, C-PH2DNP^*rf4rf4*^, C-PHPAR^*rf4rf4*^, C-PH12K5^*rf4rf4*^, and C-PH7HG^*Rf4Rf4*^, were used to test the effect of PH2F3V in different backgrounds. All of these F1s were fertile except for the F1 with C-PH7HG^*Rf4Rf4*^. B73^*rf4rf4*^ was crossed onto C-PH2F3V ^*Rf4Rf4*^ resulting in fully fertile tassels. The PHR03^*Rf4Rf4*^ F1 cross was fertile, and the PHADA^*rf4rRf4*^ F1 cross was sterile.

**Table 2:** F1 allelism crosses created with PH2F3V. F1 crosses made with PH2F3V. *Rf4* indicates restoring allele, and *rf4* indicates non-restoring allele. *Ppr153* indicates the presence of the *PPR153* gene, and *ppr153* indicates the absence of the *PPR153* gene. One dose of each gene is required for tassel fertility.

Pedigree with Rf4 and PPR153 alleles	Tassel Fertility
C-PH2DNP^*rf4 Ppr153*^/N-PH2F3V^*Rf4 ppr153*^	5
C-PHPAR^*rf4 Ppr153*^/N-PH2F3V^*Rf4 ppr153*^	5
C-PH12K5^*rf4 Ppr153*^/N-PH2F3V^*Rf4 ppr153*^	5
C-PH7HG^*Rf4 ppr153*^/N-PH2F3V^*Rf4 ppr153*^	1
C-PH2F3V^*Rf4 ppr153*^/N-B73^*rf4 Ppr153*^	5
C-PH2F3V^*Rf4 ppr153*^/N-PHR03^*Rf4 Ppr153*^	5
C-PH2F3V^*Rf4 ppr153*^/N-PHADA^*rf4 ppr153*^	1

### Chromosome 2 mapping interval contains a cluster of Rf PPR genes

High density genotyping data showed that the chromosome 2 region was the same between PH2F3V and PH269A and was inherited from their shared parent. Genome sequence data from an inbred with this chromosome 2 haplotype was used for gene content prediction. High density genotyping data showed that the restoring lines, PH2FP0 and PH480C, were the same as inbred B73 within the chromosome 2 interval. This is of interest because B73 is known to be a non-restoring inbred for CMS-C [16]. The Zm-B73-REFERENCE-NAM-5.0 genome sequence between the wide-flanking markers PZA18530 and PM01-000034U was extracted and genes were predicted using FGENESH. Using HMMer, it was determined that this QTL region contains an Rf PPR gene cluster.

This chromosome 2 PPR cluster contains five PPR genes in the PH2F3V and PH269A non-restoring haplotype, and six PPR genes in the PH2FP0 and PH480C (B73) restoring haplotype (Fig 2C). The predicted proteins encoded by these PPRs had high similarity. Amino acid sequences were 91–100% identical, and CDS sequences were 88–100% identical, excluding *PPR145* which may have an unclear gene model. Of the B73 PPRs, five had 19 repeats, one had 18 repeats. Within the smaller mapping interval, there were 23 predicted genes in total (S1 Table). Within this mapping interval PH2F3V and PH269A had two PPR genes (*PPR148* and *PPR145*) and B73 had four PPR genes (*PPR148*, *PPR153*, *PPR151* and *PPR145*) (Fig 2C).

We formed a hypothesis that the non-restoring haplotype was lacking a PPR gene required for *Rf4*’s CMS-C restoration. *PPR153* and *PPR151* were top candidate genes since they were present in the restoring haplotype but not the non-restoring haplotypes. *PPR145* was a potential candidate, as the gene model differed between the restoring and non-restoring haplotypes. *PPR148* was identical between the two haplotypes, therefore was not considered to be a candidate gene. The candidate genes for *Rf4*’s required restoration were *PPR153*, *PPR151* and *PPR145*.

### Complementation tests show PPR153 contributes to *Rf4* restoration

**Fig 3:**
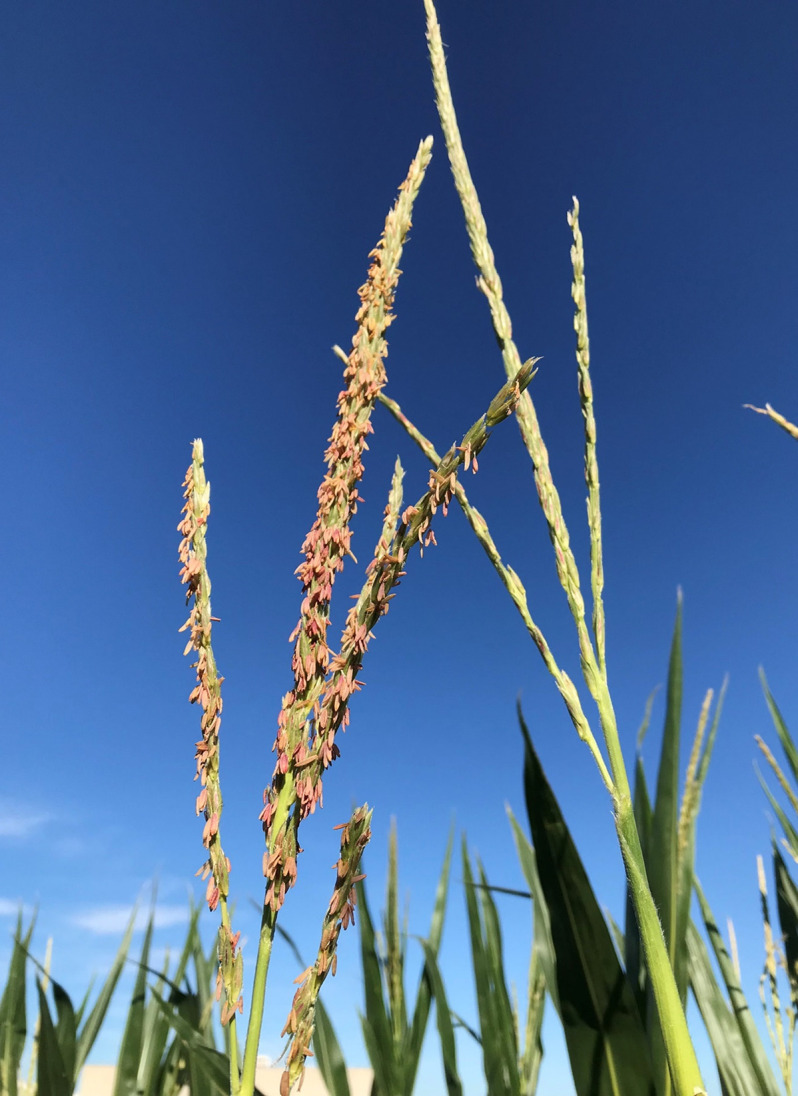
Complementation test demonstrates *PPR153* is capable of restoring CMS-C in the presence of *Rf4*. The plant on the left is hemizygous for *PPR153* gene in the C-PH2F3V^*Rf4Rf4*^ background and has restored fertility. The plant on the right is the null segregant within the C-PH2F3V^*Rf4Rf4*^ population and is sterile.

**Fig 4:**
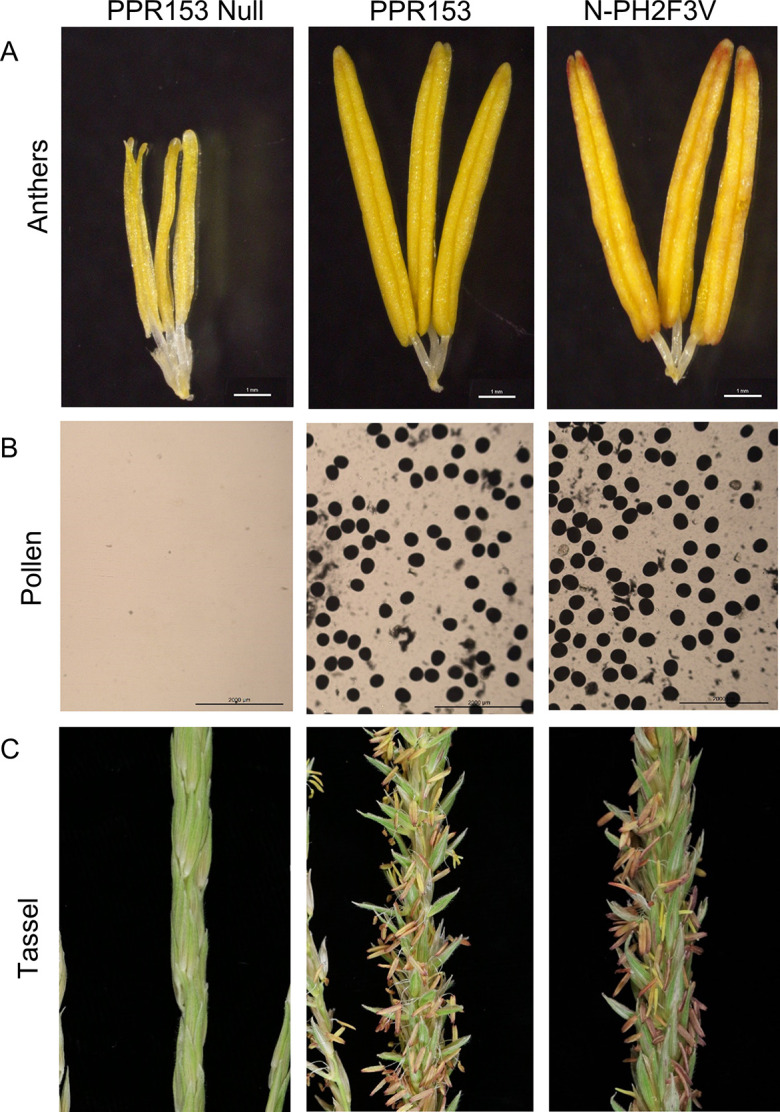
Anthers, pollen, and tassels from the *PPR153* complementation test compared to Normal fertile cytoplasm. (A) Anthers of the *PPR153* null segregants are shriveled, whereas the *PPR153* hemizygous anthers resemble Normal fertile cytoplasm anthers. (B) No pollen developed in the *PPR153* nulls, as expected from unrestored CMS-C plants. The *PPR153* hemizygous pollen and Normal cytoplasm pollen were full size and starch-filled when observed under a light microscope after 1% I_2_-KI solution staining. (C) *PPR153* null segregants had no extruded anthers, whereas the *PPR153* hemizygous and Normal fertile cytoplasm plants had extruded anthers.

Transgenic complementation was used to test the hypothesis that the non-restoring haplotype was lacking a required gene for *Rf4* restoration. The three candidate genes were transformed individually using Agrobacterium into N-PH2F3V^*Rf4Rf4*^, and then the hemizygous transgenic lines were crossed onto C-PH2F3V ^*Rf4Rf4*^. In 2022, 7 transgenic events of *PPR153*, 10 transgenic events of *PPR151*, and 8 transgenic events of *PPR145* were planted in the field. There were at least 15 plants per event for *PPR145* and *PPR151* and at least 100 plants per event for *PPR153*. The populations were segregating 1:1 for hemizygous and null plants. Every plant within the *PPR145* and *PPR151* events was sterile, demonstrating that those genes failed to complement and had no effect on CMS-C restoration. In the *PPR153* events, the hemizygous plants showed a restored phenotype, and the null segregant plants remained sterile (Table 3 and Fig 3). The *PPR153* hemizygous plants had restored anthers, pollen, and tassels resembling the fertile plants with normal cytoplasm (Fig 4). The null segregant plants’ anthers were shrunken, pollen was absent, and no anthers were exerted, as is expected from unrestored CMS-C sterile plants (Fig 4). These results show that *PPR153* (*Zm00001eb114660*) is capable of restoring CMS-C when in the presence of *Rf4*.

**Table 3:** Fertility score ratings of 7 PPR153 transgenic events. The hemizygous plants were associated with restored fertility, and the null plants remained sterile.

Event-Genotype	1	2	3	4	5	N	Mean
PPR153.39A-HEMI		3	3	21	21	48	4.25
PPR153.39A-NULL	36		1			37	1.05
PPR153.5A-HEMI	2		1	8	48	59	4.69
PPR153.5A-NULL	49					49	1
PPR153.60A-HEMI			2	10	18	30	4.53
PPR153.60A-NULL	21					21	1
PPR153.66A-HEMI		1	1	17	27	46	4.52
PPR153.66A-NULL	49					49	1
PPR153.70A-HEMI			1	7	43	51	4.82
PPR153.70A-NULL	53					53	1
PPR153.79A-HEMI			3	7	41	51	4.75
PPR153.79A-NULL	45					45	1
PPR153.95A-HEMI	2		3	6	34	45	4.56
PPR153.95A-NULL	29					29	1
						613	

*PPR153* is a gene encoding an 814-aa P-type PPR protein containing 19 repeat motifs. *PPR153* has a predicted mitochondrial cellular localization (Probability of export to mitochondria = 0.9110; MitoProt II v1.101 [17]. *PPR153* also has highest gene expression in the meiotic tassel (V18) and immature cob (V18) (https://www.maizegdb.org).

### *PPR153* is required for *Rf4* restoration in diverse NAM lines

Since the gene discovery work was performed using a small set of related germplasm, we needed to determine the role of *PPR153* in a diverse set of germplasm. The 25 NAM founder lines, previously selected to maximize diversity [18], were used to explore the *PPR153* allele frequency and function in diverse germplasm (Table 4). The chromosome 2 PPR gene cluster content was analyzed in each NAM line. Of the 25 NAM lines, six contained the *PPR153* gene with exactly identical amino acid sequence and 19 did not contain the *PPR153* gene (S3 and S4 Figs). The growing degree day heat units to pollen shedding (GDUSHD) from a Johnston, Iowa location was compiled for each line. Interestingly, the lines containing *PPR153* were in the early to mid-maturity germplasm. 78% of the NAM lines with 1550 GDUSHD or earlier contained the *PPR153* gene. No line with GDUSHD later than 1550 contained the *PPR153* gene. Using the heterotic grouping [18], none of the Tropical-subtropical NAM lines contained the *PPR153* gene.

**Table 4:** Presence of PPR153 within NAM diversity lines. For each NAM line, the presence of the PPR153 gene is indicated, and when present the gene name from MaizeGDB.org is listed. GDUSHD is growing degree day heat units to pollen shedding in Johnston, Iowa. Table is sorted by from early to late GDUSHD.

Inbred	Heterotic Group	GDUSHD	Contains PPR153	PPR153 gene IDs within reference NAM genomes)
P39	Sweet corn	1100	Yes	Zm00040ab121970
OH43	Non stiff stalk	1239	Yes	Zm00039ab115290
Il14H	Sweet corn	1286	Yes	Zm00028ab116800
MS71	Non stiff stalk	1330	Yes	Zm00035ab117760
B97	Non stiff stalk	1358	Yes	Zm00018ab117670
HP301	Popcorn	1389	No	
NC358	Tropical-subtropical	1470	No	
B73	Stiff stalk	1520	Yes	Zm00001eb114660
OH7B	Non stiff stalk	1550	Yes	Zm00038ab117050
M37W	Mixed	1645	No	
NC350	Tropical-subtropical	1645	No	
KY21	Non stiff stalk	1645	No	
CML322	Tropical-subtropical	1680	No	
CML103	Tropical-subtropical	1719	No	
CML333	Tropical-subtropical	1719	No	
CML69	Tropical-subtropical	1719	No	
TX303	Tropical-subtropical	1719	No	
Tzi8	Tropical-subtropical	1740	No	
Mo18W	Mixed	1745	No	
Ki11	Tropical-subtropical	1745	No	
Ki3	Tropical-subtropical	1745	No	
M162W	Non stiff stalk	1745	No	
CML247	Tropical-subtropical	1750	No	
CML52	Tropical-subtropical	1770	No	
CML228	Tropical-subtropical	1840	No	
CML277	Tropical-subtropical	1893	No	

A subset of 18 NAM lines were selected for further population study. For these 18 lines, the PPRCode (the 5^th^ and 35^th^ amino acid of each repeat) was extracted for each PPR gene [19], and then the homologs with identical PPRCodes were grouped. A representative gene name was assigned using NAM genome reference names from maizegdb.org. Some PPRs were nearly or completely conserved across all NAM lines, such as Zm00001eb114600 and Zm00001eb114740. Many other PPRs were uniquely found in only one NAM line, such as Zm00023ab117380, Zm00041ab118130, and Zm00028ab116940.

Populations were created for 18 NAM lines with the crossing structure C-PH2F3V^*Rf4Rf4*^ x (NAM x B73), and every plant in the population had at least one copy of restoring *Rf4* (Table 5). The population sizes were an average of 147 plants (min 119 to max 174 plants). Since the chromosome 2 region was segregating 1:1 for the NAM and B73 haplotypes, genotyping was performed with 10 markers to identify the plants with the chromosome 2 NAM haplotypes. There was at least one polymorphic marker on each side flanking the chromosome 2 PPR cluster. Only the plants with the NAM haplotypes at the chromosome 2 PPR cluster were phenotyped.

**Table 5:** NAM population phenotyping to identify restoring ability linked to the chromosome 2 PPR cluster. All plants contained restoring *Rf4*. NAM lines containing the *PPR153* gene are indicated. Only the plants containing the chromosome 2 NAM haplotypes were phenotyped for tassel fertility scores. A score of 1 to 2 is considered functionally sterile, and a score of 3 to 5 is considered functionally fertile. The percent fertile plants associated with the NAM haplotype was calculated.

NAM Inbred	Contains PPR153	Tassel Fertility Score					Total plants with NAM haplotype	% Fertile plants with NAM haplotype
		1	2	3	4	5		
OH43	Yes				3	58	61	100%
MS71	Yes			2	6	59	67	100%
Il14H	Yes	1		1	1	65	68	99%
CML333	No	1		4	6	52	63	98%
P39	Yes	1			2	46	49	98%
B97	Yes		1		1	45	47	98%
M37W	No	4		2	5	46	57	93%
Ki11	No	2	2	5	10	33	52	92%
Mo18W	No	13	8	11	5	26	63	67%
TX303	No	19	9	10	13	15	66	58%
Ki3	No	33	14	9	6	11	73	36%
CML322	No	31	5	2	1	11	50	28%
KY21	No	40	2			9	51	18%
CML103	No	39		3		1	43	9%
NC350	No	57	4	2		3	66	8%
NC358	No	41	4	2		1	48	6%
M162W	No	62		1			63	2%
CML247	No	58					58	0%

Five NAM lines contained a haplotype with the *PPR153* gene, and 98% or more of plants with those haplotypes had restored fertility, indicating *PPR153* can restore fertility in those inbred backgrounds (Table 5). In contrast, five NAM lines without the *PPR153* gene had less than 10% fertile plants, suggesting their haplotype in the chromosome 2 region is unable to restore CMS-C (Table 5). There were another five NAM populations without the *PPR153* gene with between 18 to 67% plants with the chromosome 2 NAM haplotypes having restored fertility. This partial restoration in the populations could be due to additional restorer genes segregating in the background or due to incomplete penetrance of a gene in the chromosome 2 region. Finally, there were three NAM lines, CML333, M37W, and Ki11, which had 93–98% restored fertility, which suggests they may contain a restoration factor linked to the chromosome 2 PPR cluster (Table 5). Since these three lines do not contain the *PPR153*, it is possible they contain a different PPR homolog which can function in CMS-C restoration.

## Discussion

Cytoplasmic male sterility can provide an effective and cost-efficient sterility system for commercial hybrid seed production. For wide-scale application of CMS, it is necessary to understand the genetic basis of fertility restoration to accurately predict the restoring ability of inbreds used as males and females in hybrid seed production. In cases where an inbred does not have the required restoring status, knowledge of the desirable *Rf* alleles can allow breeders to create near isogenic lines with the desirable *Rf* alleles. However, this conversion to the desirable alleles can only be successful if all the impactful restoring genes are known. In the case of *Rf4* for CMS-C, an unknown genetic factor was interfering with the restoring prediction. We sought to identify the unknown genetic factor to enable full application of the *Rf4*/CMS-C sterility system in hybrid seed production.

In this study, we demonstrated that *PPR153*, a gene encoding an 814-aa P-type PPR protein, acts in conjunction with *Rf4* to restore fertility in CMS-C plants. To begin gene discovery, the restoring trait was mapped to a 738-kb interval on chromosome 2 containing a gene cluster with six PPRs in B73. Many restorer of fertility genes are found in tandem arrays of PPR genes. PPR genes are duplicated in clusters of paralogous genes through unequal crossing over. Gene clusters can provide the source material for genes to evolve different functions, and in the case of Rf-PPRs, PPR gene clusters can lead to adaptive evolution to suppress deleterious effects of mitochondrial CMS ORFs [20,21].

The maize genome has a significant Rf-PPR cluster on chromosome 2 containing restorers for all three cytoplasm types used in commercial hybrid seed production. The *Rf3* gene is the major dominant restorer for CMS-S and is associated with the reduction in the sterility inducing transcript *orf355* [22,23]. *Rf8* and Rf* map to this PPR cluster and partially restore CMS-T when in the presence of *Rf2* [24]. *Rf8* is associated with the accumulation of the additional 1.42- and 0.42-kb T-urf13 transcripts, and Rf* is associated with 1.40- and 0.40 kb T-*urf13* transcripts [24,25]. *Rf12* is a restorer for CMS-C and maps to this cluster with at least two alleles, one of which shifts the cleavage site in *atp6c* transcripts [26,27]. *Rf12* is a dominant gene capable of restoring fertility without the presence of *Rf4*. *PPR153* also resides in this PPR cluster and is located 599-kb distal from *Rf12*. Due to the high similarity of PPRs in this cluster, it is likely these are evolutionarily homologous genes that underwent diversifying selection to adapt to new mitochondrial ORFs [20].

An analysis of this chromosome 2 PPR cluster in the NAM diversity lines shows that some PPRs are conserved in maize lines, but other PPRs are uniquely found in only one maize line (S4 Fig). In the case of *PPR153*, this gene was present at a high frequency in the early to mid-maturity lines, with 78% of the NAM lines with 1550 GDUSHD or earlier containing the *PPR153* gene. However, *PPR153* was not present in any of the NAM lines with maturity later than 1550 GDHSHD. None of the Tropical-subtropical NAM lines contain *PPR153*. *PPR153*’s allele frequency distribution in maize germplasm may have implications for the deployment of CMS-C in hybrid seed production. Inbreds in the early to mid-maturities may naturally contain *PPR153* and can be used directly in CMS-C seed production. For late-maturity, tropical-subtropical inbreds which may not contain *PPR153*, tests for restoring ability should be performed to verify the hybrid is capable of restoring CMS-C.

As was shown in this study, *Rf4* is unable to restore CMS-C alone, but instead requires an additional gene, *PPR153*. Likewise, *PPR153* is unable to restore CMS-C independently, without the presence of *Rf4*. Given the crucial role that CMS has in hybrid seed production, this discovery of the *PPR153* gene and its function in CMS-C restoration provides an important way to characterize inbreds for CMS-C sterility usage.

## Materials and methods

### Plant materials

C-PH269A^*RfRf4*^ and C-PH2F3V^*Rf4Rf4*^ are half-sib early-maturity inbreds from the stiff-stalk heterotic group and contain CMS-C cytoplasm. PH480C^*Rf4Rf4*^, PH2FP0^*Rf4Rf4*^, PH7HG ^*Rf4Rf4*^, PH1V69^*rf4rf4*^, PHADA^*rfrf44*^, PH2DNP^*rf4rf4*^, PHPAR^*rf4rf4*^, and PH12K5^*rf4rf4*^ are inbreds from the stiff stalk heterotic group. PHR03^*Rf4Rf4*^ is from the non-stiff stalk heterotic group. Both the CMS-C inbreds and normal cytoplasm inbreds were from proprietary Corteva Agriscience germplasm. For haplotype phenotyping, 18 Nested Association Mapping (NAM) diverse founder lines were acquired from the Maize Genetics Cooperation Stock Center (http://maizecoop.cropsci.uiuc.edu/).

### QTL mapping and fine mapping

In 2018 in Johnston IA, 152 plants of a C-PH269A^*Rf4Rf4*^ x (N-PH269A^*Rf4Rf4*^ x N-PH2FP0^*Rf4Rf4*^) BC_1_F_1_ population were used for whole genome QTL mapping. The BC_1_F_1_ population was phenotyped for degree of tassel fertility and genotyped with 474 polymorphic genome-wide Illumina short-read sequencing markers. QTL mapping was conducted with QTL IciMapping V3.2 (https://isbreedingen.caas.cn/software/qtllcimapping/294607.htm) using the Inclusive Composite Interval Mapping of Additive (ICIM-ADD) module. The step size was set at 1.0 cM, and the probability in the stepwise regression was set at 0.001.

In 2019, fine-mapping continued with 188 plants of the C-PH269A BC_1_F_1_ population and 291 plants of a C-PH2F3V population with a pedigree of C-PH2F3V^*Rf4Rf4*^ x (N-PH2F3V^*Rf4Rf4*^ x N-PH480C^*Rf4Rf4*^). Individual plants were phenotyped and genotyped with seven Kompetitive Allele-Specific PCR™ (KASP™) markers covering the chromosome 2 QTL interval. To genotype the fine mapping populations, KASP™ markers (S2 Table) were designed using Primer Picker software (KBioscience/LGC). To create a larger population to select recombinants, BC_1_F_1_ plants that were heterozygous in the chromosome 2 QTL interval were self-pollinated to create BC1F2 segregating seed. In 2020, 4140 kernels of the C-PH2F3V BC_1_F_2_ were genotyped with markers PZA18530 and PM01-000034G. 183 kernels with recombination events between these two flanking markers were selected for planting and phenotyping. These recombinant individuals were then genotyped with seven markers within the chromosome 2 interval.

### Tassel fertility phenotyping

The tassel fertility score of each plant was assessed every one to two days during tassel shedding using a 1 to 5 scale. If the score of a plant increased over time, the higher score was recorded. Once a plant reached a score of 5, its scoring was considered complete.

1 = No anthers extruding beyond the glumes and completely sterile.
2 = Low number of anthers exserted with some viable pollen.
3 = 1/3 of tassel exserting anthers that have normal-appearing, viable pollen.
4 = 2/3 of tassel exserting anthers that have normal-appearing, viable pollen.
5 = Whole tassel exserting anthers that have normal-appearing, viable pollen.

A score of 1 and 2 are considered functionally male sterile, and 3, 4 and 5 are considered functionally male fertile.

Pollen staining: One to three days before anthesis, anthers were collected, and fresh pollen grains were stained with Lugol’s 1% iodine potassium solution (I_2_-KI) then viewed under an optical microscope to observe starch accumulation.

### Prediction of PPR encoding genes in chromosome 2 PPR clusters

Genomic sequences of the NAM diverse founder lines and Zm-B73-REFERENCE-NAM-5.0 reference line were downloaded from NAM founder sequencing project (https://www.maizegdb.org; Portwood et al. 2019, Hufford et al. 2021). Genome sequence containing the PH2F3V and PH480C haplotype at the chromosome 2 region was obtained from Corteva Agriscience. The sequence between markers PZA18530 and PM01-000034U was extracted from all genomes. Predicted protein sequences for the longest ORF were created from FGENESH gene predictions (FGENESH algorithm with the Monocots training set; http://www.softberry.com). hmmsearch from the HMMER 3.1 package was used to screen the protein sequences for the presence of PPR motifs based on the PPR domains (PF12854, PF1304, PF13812, PF01535 and PF17177) downloaded from the Pfam database from EMBL-EBI. Predicted genes from FGENESH were compared to annotated genes presented on maizegdb.org. PPRCODE software was used to identify the 5th and 35th amino acid in each PPR motif for RNA binding sequence prediction (https://github.com/YaoYinYing/PPRCODE_Guideline; [19]. Cellular localization was predicted using MitoProt II v1.101 (https://ihg.helmholtz-muenchen.de/ihg/mitoprot.html; Claros 1996).

### Transgenic complementation tests of three candidate genes and overexpression of PPR153

Transformation constructs of the three candidate genes, *PPR153*, *PPR151* and *PPR145*, were generated by assembling synthetic DNA fragments synthesized by GenScript (https://www.genscript.com). The sequences included the gene sequence, 2-kb of native promoter sequence and 1-kb native terminator sequence. The vector contained a short primer sequence used for an assay to detect the presence of the transgene within the positive plants. Each resulting vector was introduced into N-PH2F3V^*Rf4Rf4*^ by Agrobacterium-mediated transformation. The T_0_ plants’ pollen was crossed to C-PH2F3V^*Rf4Rf4*^, and the T_1_ seed segregating 1:1 for the transgene was used for phenotypic analysis. Plants were genotyped with an assay designed to detect the primer sequence with the vector. This assay detects copy number, and the plants were either hemizygous for the transgene or null. The plants were phenotyped for tassel fertility on a single plant basis.

## Supporting information

S1 Fig LOD profile of tassel fertility scores in the C-PH269A BC_1_F_1_. (PDF)

S2 Fig Fine mapping of PPR153 using single-plant phenotyping. (PDF)

S3 Fig Amino acid alignment of PPR153 genes within NAM lines. (PDF)

S4 Fig Phylogenetic relationships of PPR sequences within chr2 PPR cluster for NAM founder set. (PDF)

S1 Table Annotated genes within the fine mapping interval. (PDF)

S2 Table Marker positions and KASP primers. (PDF)
