# Supplementary material for: Pentatricopeptide repeat 153 (PPR153) restores maize C-type cytoplasmic male sterility in conjunction with RF4
Source: PLoS One. 2024 Jul 10;19(7):e0303436. doi: 10.1371/journal.pone.0303436 (PMC11236208; doi:10.1371/journal.pone.0303436)
Supplement: S1 Fig — (PDF) [file pone.0303436.s001.pdf]

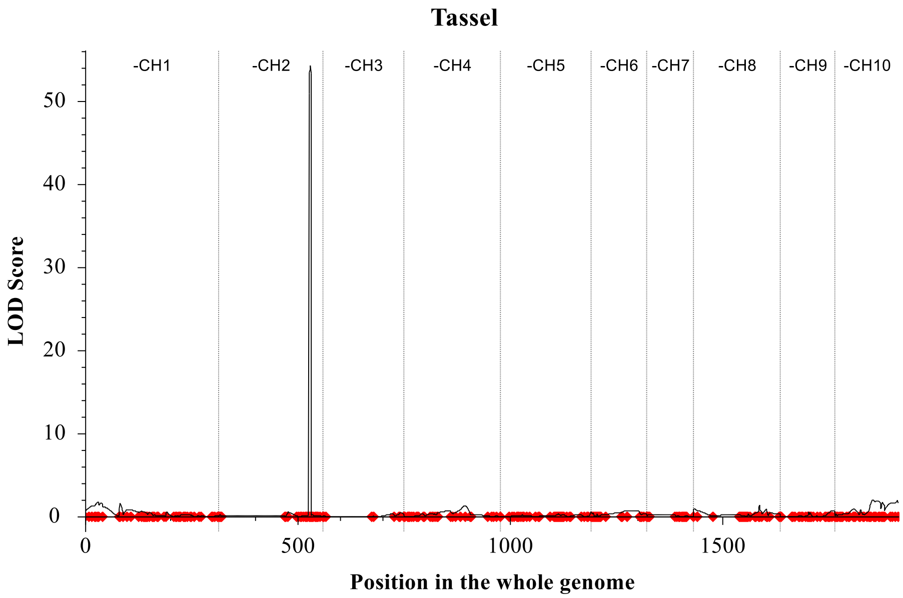

S1 Fig. LOD profile of tassel fertility scores in the C-PH269A BC_1F_1. QTL mapping performed with IciMapping V3.2 with Inclusive Composite Interval Mapping of Additive (ICIM-ADD) module show the presence of one major QTL on chromosome 2 with a LOD score of 54, explaining 81% of phenotypic variation.
