# Supplementary material for: Pentatricopeptide repeat 153 (PPR153) restores maize C-type cytoplasmic male sterility in conjunction with RF4
Source: PLoS One. 2024 Jul 10;19(7):e0303436. doi: 10.1371/journal.pone.0303436 (PMC11236208; doi:10.1371/journal.pone.0303436)
Supplement: S2 Fig — (PDF) [file pone.0303436.s002.pdf]

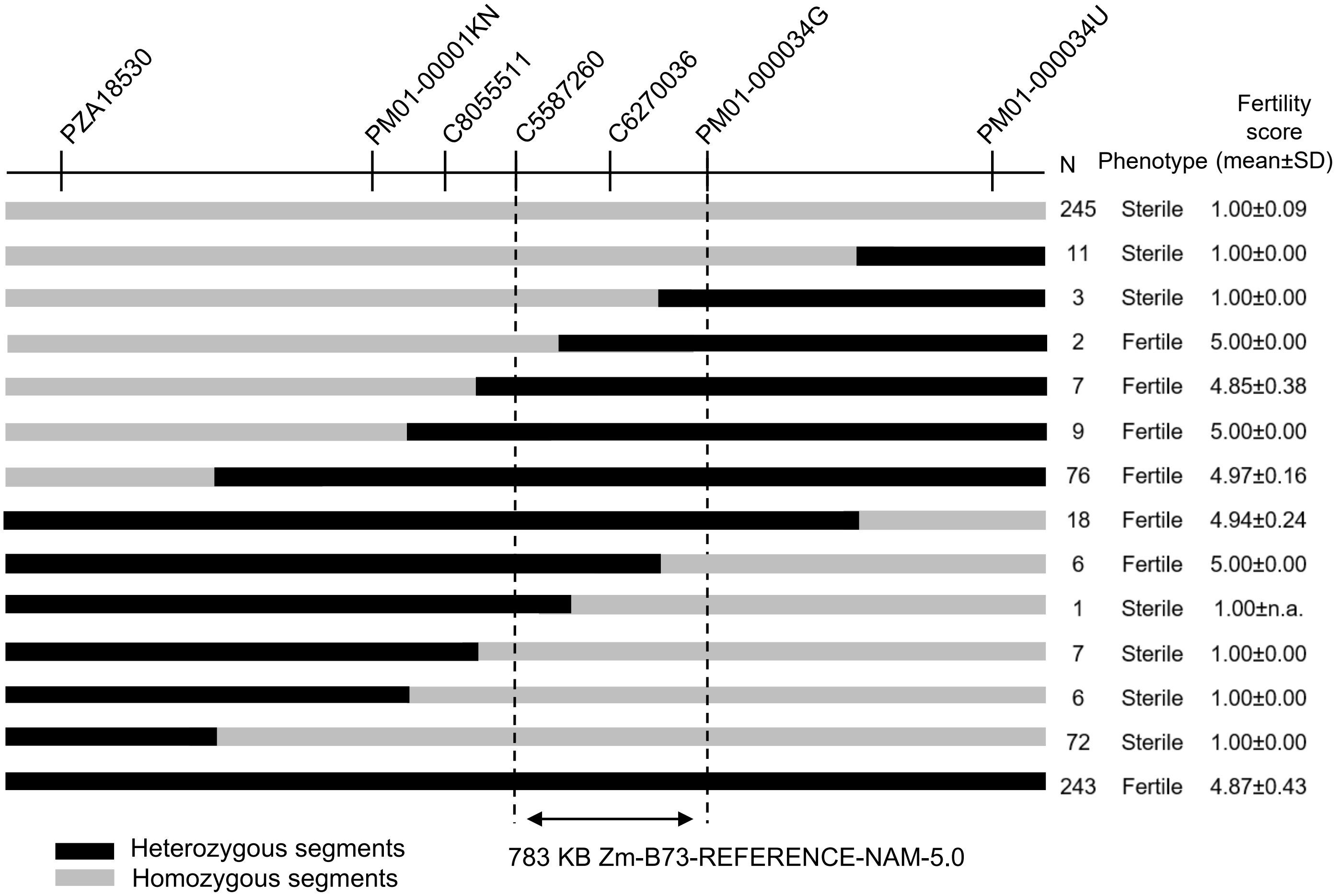


**S2 Fig. Fine mapping of PPR153 using single-plant phenotyping.** Recombinants combined from the C-PH269A and C-PH2F3V populations from the 2019 and 2020 mapping. Each recombinant type is shown with gray bars representing homozygous segments and black bars representing heterozygous segments. C6270036 was co-segregating with the phenotype.
