# Supplementary material for: Pentatricopeptide repeat 153 (PPR153) restores maize C-type cytoplasmic male sterility in conjunction with RF4
Source: PLoS One. 2024 Jul 10;19(7):e0303436. doi: 10.1371/journal.pone.0303436 (PMC11236208; doi:10.1371/journal.pone.0303436)
Supplement: S3 Fig — (PDF) [file pone.0303436.s003.pdf]

```
                               1.........11........21........31........41........51.......60
B73_Zm00001eb114660            MPSCARISSAVSTAAASSSSPPPHPPRCRRLAAATARVREGTLRPEEAHDLLDELQRRGT
B97_Zm00018ab117670            MPSCARISSAVSTAAASSSSPPPHPPRCRRLAAATARVREGTLRPEEAHDLLDELQRRGT
Il14H_Zm00028ab116800          MPSCARISSAVSTAAASSSSPPPHPPRCRRLAAATARVREGTLRPEEAHDLLDELQRRGT
MS71_Zm00035ab117760           MPSCARISSAVSTAAASSSSPPPHPPRCRRLAAATARVREGTLRPEEAHDLLDELQRRGT
OH43_Zm00039ab115290           MPSCARISSAVSTAAASSSSPPPHPPRCRRLAAATARVREGTLRPEEAHDLLDELQRRGT
P39_Zm00040ab121970            MPSCARISSAVSTAAASSSSPPPHPPRCRRLAAATARVREGTLRPEEAHDLLDELQRRGT
OH7B_Zm00038ab117050           MPSCARISSAVSTAAASSSSPPPHPPRCRRLAAATARVREGTLRPEEAHDLLDELQRRGT
                               ************************************************************

                               61........71........81........91........101.......111......120
B73_Zm00001eb114660            PVLERDLNGFLAALARAPSSAACRSGPALAVALFNRAASRAQGPRVLSPTSYTYAILMDC
B97_Zm00018ab117670            PVLERDLNGFLAALARAPSSAACRSGPALAVALFNRAASRAQGPRVLSPTSYTYAILMDC
Il14H_Zm00028ab116800          PVLERDLNGFLAALARAPSSAACRSGPALAVALFNRAASRAQGPRVLSPTSYTYAILMDC
MS71_Zm00035ab117760           PVLERDLNGFLAALARAPSSAACRSGPALAVALFNRAASRAQGPRVLSPTSYTYAILMDC
OH43_Zm00039ab115290           PVLERDLNGFLAALARAPSSAACRSGPALAVALFNRAASRAQGPRVLSPTSYTYAILMDC
P39_Zm00040ab121970            PVLERDLNGFLAALARAPSSAACRSGPALAVALFNRAASRAQGPRVLSPTSYTYAILMDC
OH7B_Zm00038ab117050           PVLERDLNGFLAALARAPSSAACRSGPALAVALFNRAASRAQGPRVLSPTSYTYAILMDC
                               ************************************************************

                               121.......131.......141.......151.......161.......171......180
B73_Zm00001eb114660            CTRAHRPELALAFFGQLLRTGLRVDIIIANHLLKGFCEAKRTDEALDILLHRTPELGCVP
B97_Zm00018ab117670            CTRAHRPELALAFFGQLLRTGLRVDIIIANHLLKGFCEAKRTDEALDILLHRTPELGCVP
Il14H_Zm00028ab116800          CTRAHRPELALAFFGQLLRTGLRVDIIIANHLLKGFCEAKRTDEALDILLHRTPELGCVP
MS71_Zm00035ab117760           CTRAHRPELALAFFGQLLRTGLRVDIIIANHLLKGFCEAKRTDEALDILLHRTPELGCVP
OH43_Zm00039ab115290           CTRAHRPELALAFFGQLLRTGLRVDIIIANHLLKGFCEAKRTDEALDILLHRTPELGCVP
P39_Zm00040ab121970            CTRAHRPELALAFFGQLLRTGLRVDIIIANHLLKGFCEAKRTDEALDILLHRTPELGCVP
OH7B_Zm00038ab117050           CTRAHRPELALAFFGQLLRTGLRVDIIIANHLLKGFCEAKRTDEALDILLHRTPELGCVP
                               ************************************************************

                               181.......191.......201.......211.......221.......231......240
B73_Zm00001eb114660            DVSSYNILLKSLCNQGKSGQADDLLRMMAEGGAVCSPDVVAYTTVIDGFFKEGDVNKACD
B97_Zm00018ab117670            DVSSYNILLKSLCNQGKSGQADDLLRMMAEGGAVCSPDVVAYTTVIDGFFKEGDVNKACD
Il14H_Zm00028ab116800          DVSSYNILLKSLCNQGKSGQADDLLRMMAEGGAVCSPDVVAYTTVIDGFFKEGDVNKACD
MS71_Zm00035ab117760           DVSSYNILLKSLCNQGKSGQADDLLRMMAEGGAVCSPDVVAYTTVIDGFFKEGDVNKACD
OH43_Zm00039ab115290           DVSSYNILLKSLCNQGKSGQADDLLRMMAEGGAVCSPDVVAYTTVIDGFFKEGDVNKACD
P39_Zm00040ab121970            DVSSYNILLKSLCNQGKSGQADDLLRMMAEGGAVCSPDVVAYTTVIDGFFKEGDVNKACD
OH7B_Zm00038ab117050           DVSSYNILLKSLCNQGKSGQADDLLRMMAEGGAVCSPDVVAYTTVIDGFFKEGDVNKACD
                               ************************************************************

                               241.......251.......261.......271.......281.......291......300
B73_Zm00001eb114660            LFKEMVQRGIPPDFVTYSSVVHALCKARAMDKAEAFLRQMVNKGVLPNNWTYNNLIYGYS
B97_Zm00018ab117670            LFKEMVQRGIPPDFVTYSSVVHALCKARAMDKAEAFLRQMVNKGVLPNNWTYNNLIYGYS
Il14H_Zm00028ab116800          LFKEMVQRGIPPDFVTYSSVVHALCKARAMDKAEAFLRQMVNKGVLPNNWTYNNLIYGYS
MS71_Zm00035ab117760           LFKEMVQRGIPPDFVTYSSVVHALCKARAMDKAEAFLRQMVNKGVLPNNWTYNNLIYGYS
OH43_Zm00039ab115290           LFKEMVQRGIPPDFVTYSSVVHALCKARAMDKAEAFLRQMVNKGVLPNNWTYNNLIYGYS
P39_Zm00040ab121970            LFKEMVQRGIPPDFVTYSSVVHALCKARAMDKAEAFLRQMVNKGVLPNNWTYNNLIYGYS
OH7B_Zm00038ab117050           LFKEMVQRGIPPDFVTYSSVVHALCKARAMDKAEAFLRQMVNKGVLPNNWTYNNLIYGYS
                               ************************************************************

                               301.......311.......321.......331.......341.......351......360
B73_Zm00001eb114660            STGQWKEAVRVFKEMRRHNILLDVVNLNTLMGSLCKYGKIKEARDVFDTMAMKGQNPNVF
B97_Zm00018ab117670            STGQWKEAVRVFKEMRRHNILLDVVNLNTLMGSLCKYGKIKEARDVFDTMAMKGQNPNVF
Il14H_Zm00028ab116800          STGQWKEAVRVFKEMRRHNILLDVVNLNTLMGSLCKYGKIKEARDVFDTMAMKGQNPNVF
MS71_Zm00035ab117760           STGQWKEAVRVFKEMRRHNILLDVVNLNTLMGSLCKYGKIKEARDVFDTMAMKGQNPNVF
OH43_Zm00039ab115290           STGQWKEAVRVFKEMRRHNILLDVVNLNTLMGSLCKYGKIKEARDVFDTMAMKGQNPNVF
P39_Zm00040ab121970            STGQWKEAVRVFKEMRRHNILLDVVNLNTLMGSLCKYGKIKEARDVFDTMAMKGQNPNVF
OH7B_Zm00038ab117050           STGQWKEAVRVFKEMRRHNILLDVVNLNTLMGSLCKYGKIKEARDVFDTMAMKGQNPNVF
                               ************************************************************

                               361.......371.......381.......391.......401.......411......420
B73_Zm00001eb114660            SYTIMLNGYATKGCLVDMTDLFDLMLGDGIAPDIYTFNVLIKAYANCGMLDKAMIIFNEM
B97_Zm00018ab117670            SYTIMLNGYATKGCLVDMTDLFDLMLGDGIAPDIYTFNVLIKAYANCGMLDKAMIIFNEM
Il14H_Zm00028ab116800          SYTIMLNGYATKGCLVDMTDLFDLMLGDGIAPDIYTFNVLIKAYANCGMLDKAMIIFNEM
MS71_Zm00035ab117760           SYTIMLNGYATKGCLVDMTDLFDLMLGDGIAPDIYTFNVLIKAYANCGMLDKAMIIFNEM
OH43_Zm00039ab115290           SYTIMLNGYATKGCLVDMTDLFDLMLGDGIAPDIYTFNVLIKAYANCGMLDKAMIIFNEM
P39_Zm00040ab121970            SYTIMLNGYATKGCLVDMTDLFDLMLGDGIAPDIYTFNVLIKAYANCGMLDKAMIIFNEM
OH7B_Zm00038ab117050           SYTIMLNGYATKGCLVDMTDLFDLMLGDGIAPDIYTFNVLIKAYANCGMLDKAMIIFNEM
                               ************************************************************

                               421.......431.......441.......451.......461.......471......480
B73_Zm00001eb114660            RDHGVKPNVVTYRTVIAALCRIGKMDDAMEKFNQMIDQGVAPDKYAYNCLIQGFCTHGSL
B97_Zm00018ab117670            RDHGVKPNVVTYRTVIAALCRIGKMDDAMEKFNQMIDQGVAPDKYAYNCLIQGFCTHGSL
Il14H_Zm00028ab116800          RDHGVKPNVVTYRTVIAALCRIGKMDDAMEKFNQMIDQGVAPDKYAYNCLIQGFCTHGSL
MS71_Zm00035ab117760           RDHGVKPNVVTYRTVIAALCRIGKMDDAMEKFNQMIDQGVAPDKYAYNCLIQGFCTHGSL
OH43_Zm00039ab115290           RDHGVKPNVVTYRTVIAALCRIGKMDDAMEKFNQMIDQGVAPDKYAYNCLIQGFCTHGSL
P39_Zm00040ab121970            RDHGVKPNVVTYRTVIAALCRIGKMDDAMEKFNQMIDQGVAPDKYAYNCLIQGFCTHGSL
OH7B_Zm00038ab117050           RDHGVKPNVVTYRTVIAALCRIGKMDDAMEKFNQMIDQGVAPDKYAYNCLIQGFCTHGSL
                               ************************************************************

                               481.......491.......501.......511.......521.......531......540
```

```
B73_Zm00001eb114660         LKAKELISEIMNNGMHLDIVFFSSIINNLCKLGRVMDAQNIFDLTVNVGLHPDDVVYSML
B97_Zm00018ab117670         LKAKELISEIMNNGMHLDIVFFSSIINNLCKLGRVMDAQNIFDLTVNVGLHPDDVVYSML
Il14H_Zm00028ab116800       LKAKELISEIMNNGMHLDIVFFSSIINNLCKLGRVMDAQNIFDLTVNVGLHPDDVVYSML
MS71_Zm00035ab117760        LKAKELISEIMNNGMHLDIVFFSSIINNLCKLGRVMDAQNIFDLTVNVGLHPDDVVYSML
OH43_Zm00039ab115290        LKAKELISEIMNNGMHLDIVFFSSIINNLCKLGRVMDAQNIFDLTVNVGLHPDDVVYSML
P39_Zm00040ab121970         LKAKELISEIMNNGMHLDIVFFSSIINNLCKLGRVMDAQNIFDLTVNVGLHPDDVVYSML
OH7B_Zm00038ab117050        LKAKELISEIMNNGMHLDIVFFSSIINNLCKLGRVMDAQNIFDLTVNVGLHPDDVVYSML
                            ************************************************************

                            541.......551.......561.......571.......581.......591......600
B73_Zm00001eb114660         MDGYCLVGKMEKALRVFDAMVSAGIEPNVVVYCTLVNGYCKIGRIDEGLSLFREMLQRGI
B97_Zm00018ab117670         MDGYCLVGKMEKALRVFDAMVSAGIEPNVVVYCTLVNGYCKIGRIDEGLSLFREMLQRGI
Il14H_Zm00028ab116800       MDGYCLVGKMEKALRVFDAMVSAGIEPNVVVYCTLVNGYCKIGRIDEGLSLFREMLQRGI
MS71_Zm00035ab117760        MDGYCLVGKMEKALRVFDAMVSAGIEPNVVVYCTLVNGYCKIGRIDEGLSLFREMLQRGI
OH43_Zm00039ab115290        MDGYCLVGKMEKALRVFDAMVSAGIEPNVVVYCTLVNGYCKIGRIDEGLSLFREMLQRGI
P39_Zm00040ab121970         MDGYCLVGKMEKALRVFDAMVSAGIEPNVVVYCTLVNGYCKIGRIDEGLSLFREMLQRGI
OH7B_Zm00038ab117050        MDGYCLVGKMEKALRVFDAMVSAGIEPNVVVYCTLVNGYCKIGRIDEGLSLFREMLQRGI
                            ************************************************************

                            601.......611.......621.......631.......641.......651......660
B73_Zm00001eb114660         KPSTILYSIIIDGLFQAGRTVPAKVKFHEMTESGIAMDICTYNIVLRGLFKNRCFDEAIF
B97_Zm00018ab117670         KPSTILYSIIIDGLFQAGRTVPAKVKFHEMTESGIAMDICTYNIVLRGLFKNRCFDEAIF
Il14H_Zm00028ab116800       KPSTILYSIIIDGLFQAGRTVPAKVKFHEMTESGIAMDICTYNIVLRGLFKNRCFDEAIF
MS71_Zm00035ab117760        KPSTILYSIIIDGLFQAGRTVPAKVKFHEMTESGIAMDICTYNIVLRGLFKNRCFDEAIF
OH43_Zm00039ab115290        KPSTILYSIIIDGLFQAGRTVPAKVKFHEMTESGIAMDICTYNIVLRGLFKNRCFDEAIF
P39_Zm00040ab121970         KPSTILYSIIIDGLFQAGRTVPAKVKFHEMTESGIAMDICTYNIVLRGLFKNRCFDEAIF
OH7B_Zm00038ab117050        KPSTILYSIIIDGLFQAGRTVPAKVKFHEMTESGIAMDICTYNIVLRGLFKNRCFDEAIF
                            ************************************************************

                            661.......671.......681.......691.......701.......711......720
B73_Zm00001eb114660         LFKELRAMNVKIDIITLNTMIAGMFQTRRVEEAKDLFASISRSGLVPCVVTYSIMITNLI
B97_Zm00018ab117670         LFKELRAMNVKIDIITLNTMIAGMFQTRRVEEAKDLFASISRSGLVPCVVTYSIMITNLI
Il14H_Zm00028ab116800       LFKELRAMNVKIDIITLNTMIAGMFQTRRVEEAKDLFASISRSGLVPCVVTYSIMITNLI
MS71_Zm00035ab117760        LFKELRAMNVKIDIITLNTMIAGMFQTRRVEEAKDLFASISRSGLVPCVVTYSIMITNLI
OH43_Zm00039ab115290        LFKELRAMNVKIDIITLNTMIAGMFQTRRVEEAKDLFASISRSGLVPCVVTYSIMITNLI
P39_Zm00040ab121970         LFKELRAMNVKIDIITLNTMIAGMFQTRRVEEAKDLFASISRSGLVPCVVTYSIMITNLI
OH7B_Zm00038ab117050        LFKELRAMNVKIDIITLNTMIAGMFQTRRVEEAKDLFASISRSGLVPCVVTYSIMITNLI
                            ************************************************************

                            721.......731.......741.......751.......761.......771......780
B73_Zm00001eb114660         KEGLVEEAEDMFSSMQNAGCEPDSRLLNHVVRELLKKNEIVRAGAYLSKIDERNFSLEHL
B97_Zm00018ab117670         KEGLVEEAEDMFSSMQNAGCEPDSRLLNHVVRELLKKNEIVRAGAYLSKIDERNFSLEHL
Il14H_Zm00028ab116800       KEGLVEEAEDMFSSMQNAGCEPDSRLLNHVVRELLKKNEIVRAGAYLSKIDERNFSLEHL
MS71_Zm00035ab117760        KEGLVEEAEDMFSSMQNAGCEPDSRLLNHVVRELLKKNEIVRAGAYLSKIDERNFSLEHL
OH43_Zm00039ab115290        KEGLVEEAEDMFSSMQNAGCEPDSRLLNHVVRELLKKNEIVRAGAYLSKIDERNFSLEHL
P39_Zm00040ab121970         KEGLVEEAEDMFSSMQNAGCEPDSRLLNHVVRELLKKNEIVRAGAYLSKIDERNFSLEHL
OH7B_Zm00038ab117050        KEGLVEEAEDMFSSMQNAGCEPDSRLLNHVVRELLKKNEIVRAGAYLSKIDERNFSLEHL
                            ************************************************************

                            781.......791.......801.......811814
B73_Zm00001eb114660         TTMLLVDLFSSKGTCREHIRFLPAKYHFLAEASP
B97_Zm00018ab117670         TTMLLVDLFSSKGTCREHIRFLPAKYHFLAEASP
Il14H_Zm00028ab116800       TTMLLVDLFSSKGTCREHIRFLPAKYHFLAEASP
MS71_Zm00035ab117760        TTMLLVDLFSSKGTCREHIRFLPAKYHFLAEASP
OH43_Zm00039ab115290        TTMLLVDLFSSKGTCREHIRFLPAKYHFLAEASP
P39_Zm00040ab121970         TTMLLVDLFSSKGTCREHIRFLPAKYHFLAEASP
OH7B_Zm00038ab117050        TTMLLVDLFSSKGTCREHIRFLPAKYHFLAEASP
                            **********************************
```

S3 Fig. Amino acid alignment of PPR153 genes within NAM lines. PPR153 aa sequence downloaded from MaizeGDB.org and aligned with ClustalW.
