# Supplementary material for: Pentatricopeptide repeat 153 (PPR153) restores maize C-type cytoplasmic male sterility in conjunction with RF4
Source: PLoS One. 2024 Jul 10;19(7):e0303436. doi: 10.1371/journal.pone.0303436 (PMC11236208; doi:10.1371/journal.pone.0303436)
Supplement: S4 Fig — (PDF) [file pone.0303436.s004.pdf]

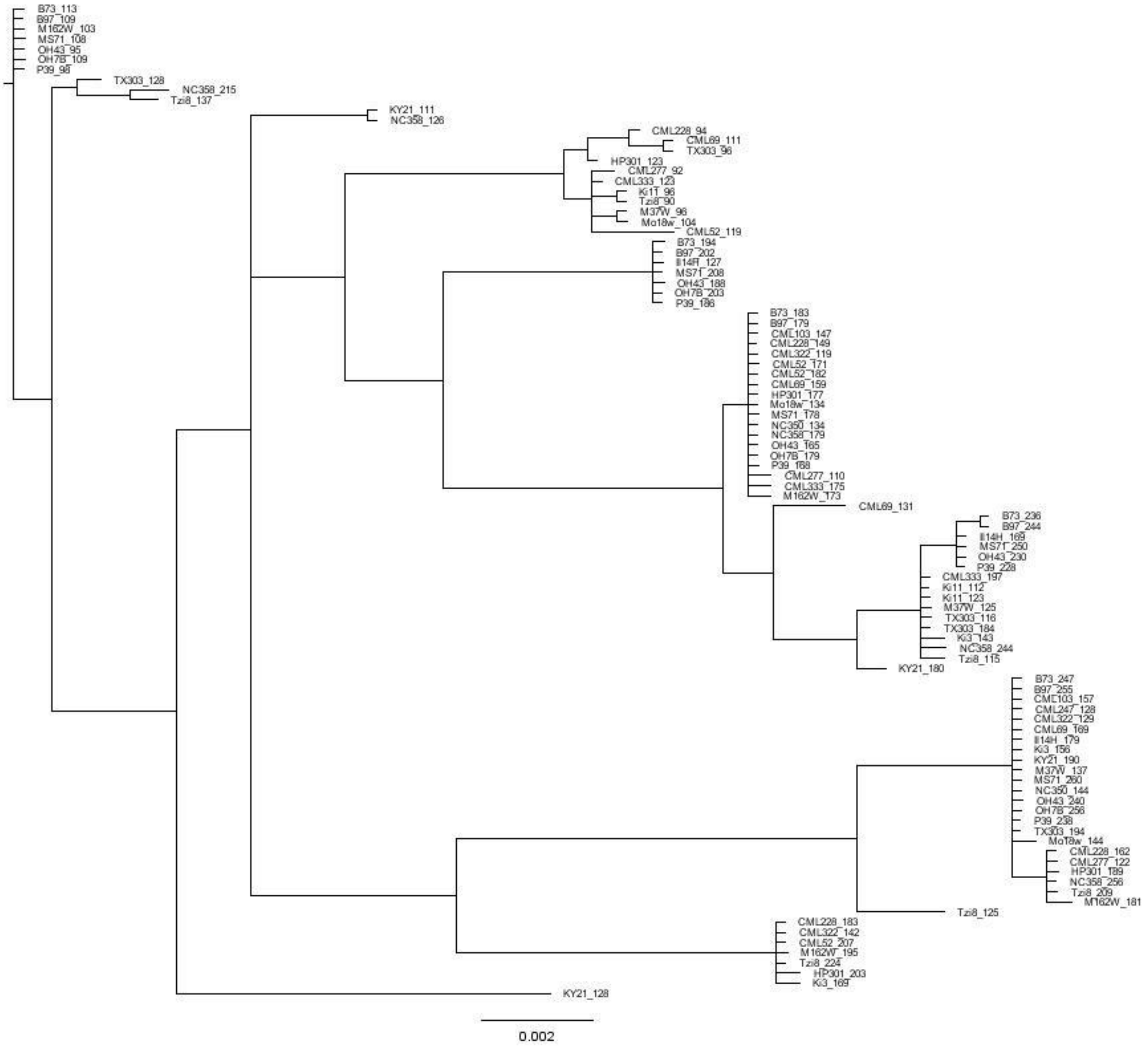

S4 Fig. Phylogenetic relationships of PPR sequences within chr2 PPR cluster for NAM founder set. The tree was generated with MrBayes 3.2.7 and visualized with FigTree v1.4.4. Gene IDs refer to the sequential gene order within the interval. B73_194 is Zm00001eb114660, B97_202 is Zm00018ab117670, Il14H_127 is Zm00028ab116800, MS71_208 is Zm00035ab117760, OH43_188 is Zm00039ab115290, OH7B_203 is Zm00038ab117050, and P39_186 is Zm00040ab121970. Results show that these 7 NAM lines contain PPR153, and PPR153 is not present within the other NAM lines.
