# Supplementary material for: Pentatricopeptide repeat 153 (PPR153) restores maize C-type cytoplasmic male sterility in conjunction with RF4
Source: PLoS One. 2024 Jul 10;19(7):e0303436. doi: 10.1371/journal.pone.0303436 (PMC11236208; doi:10.1371/journal.pone.0303436)
Supplement: S1 Table — (PDF) [file pone.0303436.s005.pdf]

S1 Table. Annotated genes within the fine mapping interval. Genes annotated within the fine mapping interval between markers C5587260 and PM01-000034G on Zm-B73-REFERENCE-NAM-5.0. PPR genes are highlighted.

Gene	Position	Protein length (aa)	Description
Zm00001eb114540	chr2:233707496..233707848	114	Putative uncharacterized protein
Zm00001eb114550	chr2:233708209..233708715	168	Galactose oxidase/kelch repeat superfamily protein
Zm00001eb114560	chr2:233733614..233734114	166	Putative uncharacterized protein
Zm00001eb114570	chr2:233786557..233787452	90	C2H2 and C2HC zinc fingers superfamily protein
Zm00001eb114580	chr2:233844246..233845254	171	Putative uncharacterized protein
Zm00001eb114590	chr2:233855115..233856636	252	GTPase activator activity; regulation of catalytic activity
Zm00001eb114600	chr2:233975324..233978256	814	ppr148 - Pentatricopeptide repeat protein148
Zm00001eb114610	chr2:233979183..233983258	260	Beta-carotene isomerase D27 chloroplastic
Zm00001eb114620	chr2:234089235..234089828	197	Putative uncharacterized protein
Zm00001eb114640	chr2:234090591..234091274	132	DNA binding;nucleosome assembly
Zm00001eb114650	chr2:234094091..234096232	171	ATP binding;protein serine/threonine kinase activity
Zm00001eb114660	chr2:234097800..234101760	814	ppr153 - pentatricopeptide repeat protein153
Zm00001eb114680	chr2:234310378..234312281	141	ATP binding;protein serine/threonine kinase activity
Zm00001eb114690	chr2:234326838..234331178	817	ppr151 - pentatricopeptide repeat protein151
Zm00001eb114700	chr2:234333870..234338031	262	DUF4033 domain-containing protein
Zm00001eb114710	chr2:234337274..234346011	200	Putative uncharacterized protein
Zm00001eb114720	chr2:234358655..234359666	57	Putative uncharacterized protein
Zm00001eb114730	chr2:234390719..234391383	154	Putative ATP synthase 24 kDa subunit mitochondrial
Zm00001eb114740	chr2:234400262..234402909	814	ppr145 - pentatricopeptide repeat protein145
Zm00001eb114760	chr2:234448440..234449985	132	Histone H2A
Zm00001eb114770	chr2:234478959..234482644	539	Hexosyltransferase
Zm00001eb114780	chr2:234484238..234490292	842	CRM family member3
Zm00001eb114790	chr2:234490799..234495361	382	CDK5RAP1-like protein
