# Supplementary material for: Pentatricopeptide repeat 153 (PPR153) restores maize C-type cytoplasmic male sterility in conjunction with RF4
Source: PLoS One. 2024 Jul 10;19(7):e0303436. doi: 10.1371/journal.pone.0303436 (PMC11236208; doi:10.1371/journal.pone.0303436)
Supplement: S2 Table — (PDF) [file pone.0303436.s006.pdf]

S2 Table. Marker positions and KASP™ Primers. SNP markers used for fine mapping with the position on the Zm-B73-REFERENCE-NAM-5.0 genome and with the KASP™ primers.

Marker Name	Zm-B73 REFERENCE NAM-5.0	KASP Primer
C5587260	233,707,873	A1: GAAGGTGACCAAGTTCATGCTACGAAGGATCAAATGCTTCCCAG
		A2: GAAGGTCGGAGTCAACGGATTCACGAAGGATCAAATGCTTCCCAA
		C1: TTGAGCACTGGGTGCATCTTGCTT
C6270036	233,981,603	A1: GAAGGTGACCAAGTTCATGCTTGGAAGCAGTTTTACTCTCGGTAC
		A2: GAAGGTCGGAGTCAACGGATTCTTGGAAGCAGTTTTACTCTCGGTAA
		C1: CATGGAAACGATTACCCGATCGAGA
C8055511	233,099,894	A1: GAAGGTGACCAAGTTCATGCTGGCATACTTGATGCCCATGTTTA
		A2: GAAGGTCGGAGTCAACGGATTCTGGCATACTTGATGCCCATGTTTT
		C1: CTGTACTAGCTACACGAATAATGGACTTT
PM01-00001KN	232,862,428	A1: GAAGGTGACCAAGTTCATGCTCAGCTTGAGCTCGAAGTTCCTG
		A2: GAAGGTCGGAGTCAACGGATTCAGCTTGAGCTCGAAGTTCCTC
		C1: GAAGGTGATATGGAGCCACCTGCT
PM01-000034G	234,491,258	A1: GAAGGTGACCAAGTTCATGCTGAAGAAGCACGTGAGCCGGCA
		A2: GAAGGTCGGAGTCAACGGATTAGAAGCACGTGAGCCGGCG
		C1: GTTGCCGTCGACGACCTGAGAA
PM01-000034U	235,820,021	A1: GAAGGTGACCAAGTTCATGCTACTTGAAATGGTCTGGGAGGCT
		A2: GAAGGTCGGAGTCAACGGATTACTTGAAATGGTCTGGGAGGCC
		C1: TTCAGGTTCCATGCAGTTGGCAGAT
PZA18530	231,435,487	A1: GAAGGTGACCAAGTTCATGCTCCTCAGTTGCTGCGCGAGC
		A2: GAAGGTCGGAGTCAACGGATTGTCCTCAGTTGCTGCGCGAGT
		C1: TGTGAAGGGCGACGATGGCCAT
